# Retinal and post-retinal contributions to the quantum efficiency of the human eye revealed by electrical neuroimaging

**DOI:** 10.3389/fpsyg.2013.00845

**Published:** 2013-11-18

**Authors:** Gibran Manasseh, Chloe de Balthasar, Bruno Sanguinetti, Enrico Pomarico, Nicolas Gisin, Rolando Grave de Peralta, Sara L. Gonzalez Andino

**Affiliations:** ^1^Electrical Neuroimaging GroupGeneva, Switzerland; ^2^Group of Applied Physics - Optics, Faculty of Physics, University of GenevaGeneva, Switzerland; ^3^Laboratoire de Neuroscience des microcircuits, EPFLLausanne, Switzerland

**Keywords:** quantum efficiency, photons, human vision, retina, dim light, scotopic vision, reaction time, EEG

## Abstract

The retina is one of the best known quantum detectors with rods able to reliably respond to single photons. However, estimates on the number of photons eliciting conscious perception, based on signal detection theory, are systematically above these values after discounting by retinal losses. One possibility is that there is a trade-off between the limited motor resources available to living systems and the excellent reliability of the visual photoreceptors. On this view, the limits to sensory thresholds are not set by the individual reliability of the receptors within each sensory modality (as often assumed) but rather by the limited central processing and motor resources available to process the constant inflow of sensory information. To investigate this issue, we reproduced the classical experiment from Hetch aimed to determine the sensory threshold in human vision. We combined a careful physical control of the stimulus parameters with high temporal/spatial resolution recordings of EEG signals and behavioral variables over a relatively large sample of subjects (12). Contrarily to the idea that the limits to visual sensitivity are fully set by the statistical fluctuations in photon absorption on retinal photoreceptors we observed that the state of ongoing neural oscillations before any photon impinges the retina helps to determine if the responses of photoreceptors have access to central conscious processing. Our results suggest that motivational and attentional off-retinal mechanisms play a major role in reducing the QE efficiency of the human visual system when compared to the efficiency of isolated retinal photoreceptors. Yet, this mechanism might subserve adaptive behavior by enhancing the overall multisensory efficiency of the whole system composed by diverse reliable sensory modalities.

## Introduction

The first experiments on the sensibility of the human eye to weak, near absolute thresholds, optical signals were conducted in the 1940s (Hecht et al., 1942). They led to the conclusion that rod photoreceptors can detect a very small number of photons, typically less than 10 during an integration time of about 300 ms (Barlow, 1956). This prediction has been confirmed by several experiments (Rieke and Baylor, 1998) making from the human eye a remarkable light sensitive detector, which can easily stand a comparison to today's best man-made detectors (Rieke and Baylor, 1998). This has even led to the proposal of using the human eye as a detector for quantum phenomena such as entanglement (Sekatski et al., 2009).

The quantum efficiency (QE) of the human eye as a detector, i.e., the probability of getting a response given that a photon impinges on the cornea, has been determined using two different approaches: behavior and direct neural recordings. In behavioral terms the QE can be estimated from the frequency of seeing curves (FoS) (Hecht et al., 1942) later replaced by a distribution of ratings (Sakitt, 1972). Flashes of light, with a controlled probabilistic distribution of photons are sent into the pupil and subjects who are dark adapted are prompted to indicate if they perceived a flash. The detection threshold, i.e., the number of photons required to trigger a conscious percept (arbitrarily defined as the light intensity giving rise to 60% detection), is determined by measuring the fraction of trials in which a flash is reported as perceived as a function of the number of photons incident at the cornea. Since, only about 8% of the photons incident on the cornea reach the retina, hence about 100 photons are required to trigger a neural response even if rod photoreceptors can react to single photons (Rieke and Baylor, 1998).

Direct neural recordings have been used in toads (Baylor et al., 1979) and monkeys (Baylor et al., 1984) to determine QE from the number of photons needed to evoke responses in isolated rod photoreceptors. These studies lead to the conclusion that rod photoreceptors can signal the absorption of single photons. Consequently, estimates of QE vary in about one order of magnitude as a function of the definition of response (neural response in photoreceptors vs. behavioral responses) used for its quantification. Behavioral measurements based on the FoS curve place the QE of the human eye between 0.03 and 0.06 while direct estimates based on losses within the eye range from 0.1 to 0.3 (Baylor et al., 1979). Consequently, the QE estimated from behavior is very low compared with the absorptive QE estimated from the properties of light photoreceptors at the retina.

The reasons for the divergence in the estimated and measured QE are not completely clear as the processes limiting sensitivity are not yet fully characterized. When estimating QE from the FoS curve we demand to the observer to indicate whether or not they perceived the stimuli. According to classical psychophysical models (Krantz, 1969), this detection process is composed of at least two psychological components or processes: (1) the sensory process transforming the physical stimulation into internal sensations and (2) a decision process which decides on responses based on the output of the sensory process. Each of the two processes is, in turn, characterized by at least one parameter: the sensory process by a sensitivity parameter and the decision process by a response criterion parameter. To avoid confounding the sensitivity of the sensory process with the response criterion of the decision process, one needs to measure two aspects of detection performance: the conditional probability that the observer says “yes” when a stimulus is present (the hit rate, or True positive rate closely linked to the FoS curve) but also the conditional probability that the observer says “yes” when the stimulus is not present (False positive rate or FAR).

Barlow (1956; Hallett, 1969) relied on the concept of false positive rates to explain the discrepancy in QE. He attributed the fact that observers occasionally report seeing a flash even when no light was delivered to the existence of what he termed “dark light” or “dark noise (DN).” Indeed, if there were no DN, there would be no reason why a single photon should not be seen as rods reliably signal the absorption of single photons across repeated trials (Rieke and Baylor, 1998). Behavioral sensitivity and dark noise can be the result of Poisson fluctuations in photon absorption at the level of the retina. Experiments in toads have shown that one of the possible source of this dark-noise is the thermal ionization of the photosensitive protein in the retina, pointing that sensitivity of frog was decreasing with temperature (Aho et al., 1993). Upon this model, dark noise increases the rate of false-positive affecting the sensitivity of the detection threshold and the reliability of the QE estimated from behavior. Indeed, if the threshold of vision were set to a single photon, there would be a false alarm rate since the threshold criterion would be exceed by the DN in the absence of stimulation. Consequently, observers need to adjust the threshold to minimize false percepts (false positive rate).

Is retinal noise the only factor impacting the response criterion that characterizes the decision process? If this were the case then retinal (dark) noise would be the only explanation for the observed discrepancy in QE and the only factor limiting sensitivity in visual perception. Yet, noise is not an exclusive property of retinal photoreceptors. Noise might arise anywhere into the chain of neural processing and add to fluctuations at the level of the retina. Supporting the existence of post-retinal contributions to dark noise are the experiments reporting perception of phosphenes after Transcranial Magnetic Stimulation (Romei et al., 2008) at the level of the occipital cortex. Some of these studies indicate that the phosphene perception appears after extensive recurrent processing and is therefore not purely attributable to primary visual processing (Taylor et al., 2010). In addition, deciding that the stimulus is present or absent is clearly not a matter of sensory evidence alone as a decision about stimulus absence lacks sensory evidence by definition. Interestingly, cells coding for the decisions about both, the absence and presence of stimulus, have been recently reported at the prefrontal cortex of primates (Merten and Nieder, 2012) and decision cells found at the parietal cortex (Shadlen and Newsome, 2001). Noise at the level of prefrontal and parietal circuits might equally impact decisions contributing to the discrepancy in QE. Finally, the state of post-retinal networks at the time (or even before) the stimulus impinges the retina seems to play a major role on its conscious detection (Busch et al., 2009). Consequently, pre-stimulus states might limit the sensitivity and QE of human vision despite being a process completely independent of retinal photoreceptors. The impact of such post-retinal mechanisms on the reduction of QE remains unknown.

To shed further light on the post-retinal mechanisms impacting the QE of the human eye we repeated a version of the FoS experiment described in Hecht et al. (1942). We hypothesized that post-retinal processes substantially contribute to the observed decrease in QE when inferred from the FoS curve. We adhered to the only accessible measure of QE we can rigorously control in this experiment, i.e., the fraction of incident photons that contribute to conscious perception as measured by the FoS curve. Besides psychophysics and signal detection theory, we relied on two other complementary methodologies helpful to dissociate the stages of processing impacting sensitivity. First, reaction times (mental chronometry) which allows inferring to some extent the content, duration, and temporal sequencing of cognitive operations within perceptual processes (Sternberg, 1969; Jensen, 2006). Second, scalp measured Event related Potentials (ERPs) which provide an indicator of the latency of neural responses at the different processing stages (Thorpe et al., 1996).

## Materials and methods

### Recording protocol

#### Participants

Twelve healthy young volunteers (age range: 26–38, mean age 30 ± 4 *SD*, 2 females) were recruited from the faculties of Physics and Medicine of the University of Geneva. Eleven of the participants were right-handed. They had no history of neurological problems. The whole experiment was approved by the local ethics committee (Geneva University Hospitals). Participants were verbally informed of the goals of the experiment and the sequence of events.

#### Dark adaptation

The experiment was carried out in a completely dark recording room with all potential sources of light, e.g., computer LEDS, covered by black plastic tape. Participants were dark-adapted before the experiment by being kept during 40 min in the dark room while wearing a black sleeping mask. The darkness was kept during the approximately two hour's duration of the experiment.

#### Rationale of the experiment and instructions to participants

In the absence of invasive neural recordings we don't have direct access to the sensory and decision processes we would like to disentangle. Nevertheless, we can extract inferences on the contribution of retinal and post-retinal processing through the analysis of observable indirect measures of neural activity such as the EEG or from behavior (for example signal detection theory and RTs).

Two crucial aspects to consider when trying understanding QE are: (1) the origin of the systematic variability in a subject's perceptual outcomes exhibited across trials sharing identical stimuli, and (2) the variability across different observers. For instance, Hecht et al. (1942) and Van Der Velden (1946) estimated the detection threshold and QE directly from the FoS curve. Their approach relied on the assumption that variability in a subject's responses is due to the Poisson statistics of photon absorption and the consequent trial-to-trial fluctuations in the number of photons absorbed (Field et al., 2005) by the retina. Yet, according to Rieke and Barlow (Rieke and Baylor, 1998) rod's elementary responses to single photons are highly reproducible across trials and estimates of Dark Noise are very low. However, the variability across trials within the same human observers is very high under dim light conditions. If the reproducibility of the elementary response allows the number of photons absorbed to be estimated accurately from the rod's response what are the causes for such a large inter observer variability? Could trial-to-trial fluctuations in perceptual outcome (Busch et al., 2009; Mathewson et al., 2009; Vanrullen et al., 2011) or in Response Times (Gonzalez Andino et al., 2005) be influenced by trial-by-trial fluctuations in the state of the attentional networks at the time of/before stimulus onset? Note that attentional fluctuations during pre-stimulus states can be assessed by the power or phase of ongoing pre-stimulus oscillations recorded by scalp EEG contacts. Importantly, if the variability in sensitivity across trials were only a function of the statistics of photon absorption at the retinal level we should observe no statistically significant differences in neural signals between seen and unseen trials over the pre-stimulus period. The analysis of the EEG signals over the pre-stimulus period can be therefore used to shed light on non-retinal contributions to decreases in sensitivity under dim light conditions.

Substantial gaps are seen between the best possible performance of an ideal observer near the visual threshold and the actual performance of a human subject (Packer and Williams, 2003). Even after taking all of the optical losses into account human subjects are significantly below the ideal observer performance with some healthy young subjects performing worse than others despite no obvious visual deficits. Across subject variability has been attributed to changes in decision criteria, a concept developed within signal detection theory. Indeed, the experiments of Sakitt and Barlow (Barlow, 1956; Sakitt, 1972) show that false positives can trade for detection threshold across a wide range of criteria which are independent of the intensity of the stimuli. In this view, different criteria correspond to different signal-to-noise ratios, and observers choose where to operate on the basis of how many mistakes they are allowed to make. These additional losses, i.e., missed trials in subjects which adopt a conservative decision threshold to minimize false positive trials, must be attributed to neural processing as decisions are not taken at the retinal level. The phenomenon is reminiscent of the so called speed–accuracy trade-off (SAT) and has been observed in many decision-making tasks (Fitts, 1966; Woodworth, 1899). Two alternative explanations to the SAT have been put forward: (1) *the response threshold account* that hold that fast responses occur when response thresholds are too low and not enough sensory information has been accumulated to support an accurate judgment (Ratcliff, 2002; Bogacz et al., 2006). On this account, the SAT is mediated by late-stage decision processes happening just before the initiation of motor responses and (2) *the sensory-readout hypothesis* which attributes the changes in decision criteria to the efficiency with which sensory evidence is accumulated during decision making (Ho et al., 2012). On this account performance decrements may result from a failure to optimally process sensory signals. For example, the failure to optimally read-out sensory signals in our experiment might be a consequence of a failure to encode or to keep in working memory briefly presented stimuli due to fluctuations in pre-stimulus attentional states. Investigating RTs and neural correlates of sensitivity across subjects differing in decision criteria might shed further light on the origins of variability in perceptual thresholds.

By combining information from RTs and the latency and scalp positions of peaks in the ERP signal we expect here to extract information on the relative timing of the sensory and decision processes. According to previously described decision making models, decisions occur when neural signals reach a certain threshold. Noise will alter the gradual accumulation of neural information speeding up or retarding the decision time. Reaction times (RTs) therefore correlate with the time needed to reach the threshold that is dependent on the difficulty of the choice (Smith and Ratcliff, 2004) as well as from other factors. Weak or uncertain stimuli lead to slowly varying accumulation of evidence and longer decision times while certain/strong stimuli lead to quickly growing accumulation of certitude that is reflected in a sharp buildup of neural activity that quickly reaches the necessary threshold to reach decisions speeding up the RTs. On this basis we should expect: (1) an inverse dependency between RTs and the intensity (number of photons) of the flashes, (2) Significant differences between mean RTs corresponding to different stimulus intensities.

The instruction given to the subjects was explicit: “report seeing the flash via a button press when you feel completely confident about the percept.” We avoided a multiple choices task reflecting the trial by trial confidence in perception (Barlow, 1956) and Sakitt (1972). We did so on three bases: (1) RTs are known to vary as a function of the difficulty of the task. Yet, the difficulty of the task is not only linked to the perceptual difficulty that we want to investigate here but also to the number of choices available. There is ample experimental evidence supporting the increase in RTs with the number of available choices (Hick, 1952; Usher et al., 2002), at least for untrained subjects (Mowbray, 1960). Since we were interested in the link between perceptual processes and RTs rather than on the link between choices and RTs we considered the two choices alternative as the most reasonable one. (2) The number of errors is known to increase with number of choices as repeatedly shown in the literature (Teichner and Krebs, 1974). This is sometimes due to false button presses. Since choosing between several buttons in full darkness is more challenging than under normal illumination conditions then the probability of false button presses increases. Consequently, the two choices task adopted here is “optimal” to: (i) isolate real “dark noise” coming from retinal/post-retinal effects from motor mistakes and (ii) to isolate the perceptual component of the RTs from the choice component. (3) Subject's performance and EEG signals tend both to worsen with the duration of the experiments. As previously explained, multiple choices tasks lengthen the experiment. A condition for the experiment is to remain attentive and still as to obtain adequate signal to noise ratios in EEG signals and sustained performance. This posed a challenge to some of the participants as the full experiment lasted for ~2.30 h.

#### EEG recordings

The scalp electroencephalogram (EEG, 64 channels) and RTs were recorded during the experiment. The EEG was recorded at variable frequency sampling (1024 or 2048 Hz) to guarantee the temporal precision of the triggers and response time. Frequency sampling was individually selected on the basis of the initial psychometric curves. Recordings were done using the Biosemi system with 64 sintered Ag-AgCl electrodes and implicit filter settings at 5th order sinc filter with a −3 dB point at 1/5th of the sampling frequency. The electrodes were mounted on the manufacturer-provided cap according to an extended 10–20 system. The Biosemi system uses a common mode sense (CMS) active electrode as the reference and fully DC coupled amplifiers. Visual inspection was used to reject artifact-contaminated trials. Bad electrodes interpolation was based on spherical splines using Cartool. Epochs of 2000 ms (one second before the presentation of the stimulus) were extracted after notch filtering at 50 Hz and superior harmonics. Baseline correction was based on 200 ms prestimulus window.

#### Experimental setup

**Figure 1:**
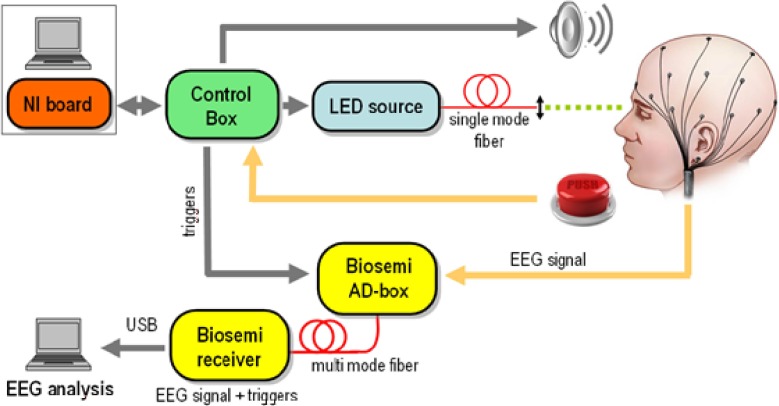
**Experimental Setup**. A light emitting diode (LED) was digitally controlled to produce pulses of light at 500 nm wavelength with power varying between 8 and 400 pW and very short duration (between 100 and 1 ms depending on the subject). The power of the light was individually adjusted to each participant using gray filters and sent through collimation lens at the end of the fiber to optimally focus the beam to form an angle of ~20 degrees with respect to the eye's axis retina where the density of rods is higher. EEG recordings, done using the Biosemi system, were synchronized with the beams onset at the μs level. Around 180 repetitions of four different intensities of light were randomly presented to each participant. In additions an equivalent number of trials (180) were included were participants received a warning signal but no light beam was actually sent into the retina.

The experimental design is schematically depicted in Figure 1. A light emitting diode (LED) was used to produce flashes of light at 500 nm wavelength which guarantees maximum sensitivity of rod cells (Alpern, 1987). A portion of the light was collimated and coupled into a single mode fiber. This kind of optical source was chosen for safety reasons as only a maximum power of hundreds of pW can be coupled into the fiber. The LED was software controlled via a National Instruments digital to analog card that allows varying the power of each light pulse between 8 and 400 pW, while its duration can be chosen between 100 μs and 1 ms. In this way, the number of photons in each pulse can be dynamically varied by nearly three orders of magnitude. We used neutral density filters (gray filters) to further decrease the optical intensities by a factor *t*_*ND*_ and adapt them to the subject's sensitivity that was individually detected as described in next section. While *t*_*ND*_ changes from subject to subject it is set to at least 0.1.

**Figure 2:**
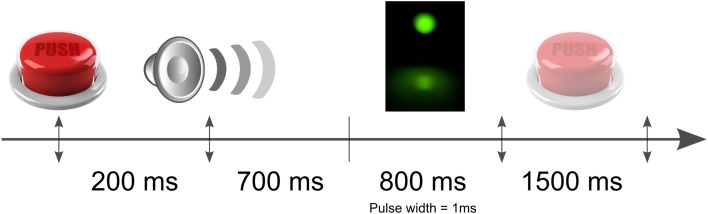
**Schematic illustration of the time course of the experiment**. The light pulses were equally randomly distributed within a time windows of 800 ms after the auditory signal. The subjects had 1500 ms to indicate by a button press if he perceived or not the flash.

The light coupled into the fiber was directed to one eye of the subject, who rested his chin and forehead on a chinrest support to keep the head steady over the experiment. A collimation lens at the end of the fiber allows focusing of the beam on the retina. Since the density of rod cells (the most sensitive human photo-receptors) is highest in the peripheral region of the retina, the direction of the beam is chosen to form an angle of approximately 20 degrees with respect to the eye's axis. The retina is illuminated on the temporal side, to avoid the optical nerve. The photons emitted by the LED are Poisson distributed. At least 700 ms before the light is emitted, an acoustic signal is produced to alert the subjects of the imminent emission of the pulses. The subjects press a button in case of conscious perception of the flash and a digital signal is sent to the NI board. The communication with the board is managed via a control box. Finally, the control box sends to the Biosemi AD-Box the trigger signals corresponding to (1) the timing of the acoustic signal, (2) the value of the randomly chosen intensity and (3) the timing of the button press for perceived flashes. At least 150 repetitions of each intensity and the same amount of blank trials (the acoustic signal is given but the flash is not sent) were obtained per each subject. The time course of the whole experiment is shown in Figure 2.

#### Estimating the number of photons incident at the cornea as a function of the power emitted by the LED.

The power emitted by a LED is linear with respect to the voltage bias applied to it and linearity was assessed in our case to hold up to 9 Volts. A calibrated power meter was used to measure the power *p* which exits the fiber. The number of photons per second corresponding to the power measured when the LED is at 9 V is given by:

(1) $$n^{*}=\frac{P\lambda }{hc}$$

where λ is the wavelength, *h* is Planck constant and *c* is the speed of the light. For each subject 4 different intensities were individually selected by varying the voltage applied to the LED from the *V*_offset_ ≈ 2.5 V to 9 V. As the power emitted by the LED is linear between 2.5 and 9 V, the value of *P* in Equation (1) must be multiplied by a factor *t*_bias_ = (*V*_*I*_ − *V*_offset_)/(9*V* − *V*_offset_), where *V*_*I*_ the voltage is applied to the LED for the specific intensity *I*.

The fact that the gray filters reduce the power by a factor *t*_*ND*_ must be taken into account into the calculations as well as the fact that the pulses had a very short duration Δ*t*_pulse_ that varied according to the subject from 0.5 to 1 ms. Therefore, the final formula used to compute the number of photons at each pulse that were sent to the cornea is finally given by:

(2) $$n_{\text{pulse}}=\frac{t_{\text{bias}}\times t_{ND}\times \Delta t_{\text{pulse}}\times P\times \lambda }{h\times c}$$

#### Selection of the best individual parameters (method of adjustment)

Once subjects were adapted to darkness and the EEG set up installed we carried out some initial tests to individually tune the attenuation of light (achieved by the gray filters) and the duration of the pulses. Several attenuations were tested for each of the four intensities and subjects were requested to report if they perceived the flashes. The final attenuations were chosen as those for which the flashes were perceived in half of the presented pulses. This approach was preferred to the on-line selection of the intensities in order to minimize the duration of the experiment. Finally, during the experiment, four intensities of flashes were presented to each subject with attenuations chosen to evoke perception in half of the trials. In what follows, we will use the term intensity for the intensity of the beam reaching the cornea, i.e., after the filters.

### Data analysis and rationale

#### Reaction time analysis

We investigated if and how RTs, defined as the time elapsed between the onset of the flash and the button press indicating perception, vary as a function of (1) the number of incident photons, and (2) the accuracy of decisions.

RTs were compared using the One-Way ANOVA if data had a normal distribution (according to the Lilliefors test, matlab statistical toolbox, 0.05 significance level) or using the Kruskal-Wallis test (non-parametric One-Way ANOVA) for non-normally distributed data. Unless otherwise specified, all the statistics and analysis were done using Matlab R2006a. When appropriated, we display behavioral data using notched boxplots that provide a summary of several important features of the distribution of values (e.g., median, confidence interval around it, outliers). In these plots, when the notches of two or more groups do not overlap then the medians of the two groups differ at the 5% significance level.

#### Frequency of seeing (accuracy) and QE

The 4 pre-selected intensities and the blank trials were presented to the subjects in random order with each intensity repeated at least 150 times. The frequency of seeing curve was obtained plotting the proportion of flashes reported as perceived as a function of the intensity. The energy of the flashes was transformed into the average number of photons using Equation (2).

The probability of seeing decrease with the decrease in the number of presented photons. However, determining which threshold should be used to determine the minimal number of photons necessary to generate conscious detection is still an open question. Hecht et al. set the threshold at 60% of probability and concluded that 54–148 incident photons are needed to trigger conscious detection. Here, we decided to set a slightly lower threshold at 50%. This is due to three reasons, (1) we introduced under the form of zero intensity trials a control against dark noise, i.e., detection by chance and (2) we used several naïve (untrained) subjects and (3) the experimental design introduced a variable delay between the acoustic signal and the photons arrival to prevent anticipation.

In order to more precisely estimate the number of flashes absorbed by the retina that are necessary for conscious perception from discrete observations, the probability of seen curve is typically fitted with a model. For instance, Rieke and Baylor (1998), following Hecht et al. (1942), made the assumption that for a given intensity the number of photons absorbed by the retina follows a Poisson-distribution. In Rieke's model, the probability of seeing a flash (*p*_see_) of intensity *I* can be written as:

(3) $$p_{\text{see}}\left(I\right)=\sum_{n=\theta }^{\infty }\frac{e^{\left(-\alpha I\right)}}{n!}{\left(\alpha I\right)}^{n}$$

Where θ is the minimal number of photons, under which the subject never perceived a flash and α represents the decrease in intensity between the number of photon sent in the flash and the amount of photons arriving at the retina. The value α × *I* represents thus the mean number of photon absorbed by the retina.

However, after extensive testing on the data we found that much better fits are obtained assuming a log-poisson regression model distribution, i.e.,

(4) $$p_{\text{see}}\left(I\right)=\sum_{n=\theta }^{\infty }\frac{e^{\left(-\log\left(\alpha I\right)\right)}}{n!}\log{\left(\alpha I\right)}^{n}$$

The free parameters θ and α were therefore determined by the simultaneous minimization of the objective function *f* given by:

(5) $$f\left(\theta ,\alpha \right)=\sum_{k=1}^{4}{\left[p_{\text{see}}\left(I_{k}\right)-\sum_{n=\theta }^{\infty }\frac{e^{\left(-\log(\alpha I_{k})\right)}}{n!}\log{\left(\alpha I_{k}\right)}^{n}\right]}^{2}$$

Where *I*_*k*_ are the four intensities presented to each subject. To perform the minimization, we arbitrary selected a range of possible values for α and θ and computed the function for all the possible pairs of parameters. Note that the fits are here used to get an adequate approximation of the number of photons needed for conscious detection at the 50% probability. By no way, should these fits be considered as a model for the detection probability as they have been obtained from just four intensities.

#### Measuring sensitivity using signal detection theory: the sensitivity index (d′)

A criticism to the FoS curve as a measure of sensory sensitivity is that it mixes the decision criteria with sensory sensitivity. We therefore used as an additional measure the sensitivity index (d′) which splits detection performance into two components: the conditional probability that the observer says “yes” when a light is present (the hit rate or true positive rate TPR) and the conditional probability that the observer says “yes” when a light is not present (the false alarm rate, or false positive rate FPR) (Green and Swets, 1966). We computed the sensitivity index d′ as:

$$d^{\prime }=z(\text{TPR})-z(\text{FPR})$$

where *z*(·) denotes the inverse cumulative normal distribution of the given probabilities, FPR is the false-positive rate and TPR is the true-positive rate. For subjects showing perfect specificity (FPR = 0), since *z*(0) = ∞, we replaced FPR by 1/*N*, with *N* denoting the number of trials.

A true positive (*TP*) is a non-zero intensity trial reported as perceived while a true negative (*TN*) is a non-perceived zero intensity trial. A false negative (*FN*) is a non-zero intensity trial that is non-perceived and a false positive is a zero intensity trial reported as perceived. The *TPR* and *FPR* are, respectively, defined as:

(6) TPR=TP/(TP+FN)

(7) FPR=FP/(FP+TN)

#### EEG analysis (event related potentials, ERPs)

Two categories of trials were analyzed: *the hits*, i.e., trials were real flashes were sent and were reported as perceived and *the misses*, i.e., trials were flashes were sent but reported as non-perceived. ERPs were computed by averaging the epochs on each category aligned by the onset of the flash for every subject and intensity. Both, the original Biosemi reference and the average reference were used in all the analysis to assess independence of the effects on the chosen reference. Note that the reference removes the effect of a constant from the data. The Grand Mean (GM) was afterwards computed as the average over subjects of the individual ERPs once normalized by the norm of the global scalp energy. This normalization avoids overweighting the contribution of individual subjects to the GM due to the interindividual variance in baseline EEG power obeying to different geometrical factors (e.g., skull thickness).

To determine the presence or absence of ERP responses at both the single subject/single electrode traces and at the single electrode/GM data we computed baseline adjusted *z*-scores. A time interval at a single electrode was defined as showing a consistent ERP response if its absolute amplitude deviated by more than 2.5 SD from the mean amplitude measured in the 200 ms baseline period (*z*-score > 2.5, *p* < 0.01).

To shed light on the neural substrates of across-subjects variability we performed two different analyses. First, we compared the ERP responses across two groups of observers who differed in terms of their false alarm rates (dark noise). Following Barlow (1956) and Sakitt (1972), differences in the false alarm rate should reflect differences in the decision criteria adopted by the observer across the experiment. Consequently, if the decision criterion is already set during retinal processing, we should observe early ERP differences between the groups of observers who rely on different criteria. If, on the contrary, the decision criteria is set later in the neural transduction chain we should observe differences in the later ERP components. Our second analysis was aimed to detect the scalp location and timing of ERP components correlating with sensitivity across subjects. If individual sensitivity is purely determined by retinal processing then one should expected a high correlation already early in time (endogenous ERP components) and for scalp contacts covering the occipital cortex. If, on the other hand, extra-retinal factors contribute to variations in sensitivity across subjects then correlations should appear later in time being closer to decisions and therefore to the RTs. In later case correlations are expected to be maximal over frontal/parietal contacts which are areas assumed to contain decision related cells. We computed, across subjects, the Spearman's rank correlations between the sensitivity and the single electrode ERP traces on a frame by frame basis over the 1200 ms period covering the 200 ms baseline and the one second after stimulus onset. The significance of the correlation was computed using the exact permutation distribution and correction for multiple tests based on the “dunn-sidak” approach.

#### EEG analysis (prestimulus states)

To investigate the alleged role of pre-stimulus states on temporal fluctuations of the visual detection threshold we compared hits and misses trials at the single electrode level in the time-frequency domain The analysis approach is indeed very similar to the one used by Busch et al. (2009) to demonstrate the temporal fluctuations of the visual detection threshold along with the phase of ongoing EEG activity in a similar task. We computed the Stockwell transform (ST) of each single-trial EEG data for the frequency range from 0.4 to 130 Hz (frequency resolution of 0.4 Hz) and for the temporal window from −1.5 to 1 [s] around the stimulus. The ST (S-transform), developed in 1996 for analyzing geophysics data [2], is a modification of the Continuous Wavelet Transform. In contrast to the Wavelet Transform, which describes signals in terms of scales and dilations, the S-transform deals directly with time and Fourier frequencies.

Since the goal of this analysis was to investigate the sources of trial by trial variability in visual detection threshold rather than interindividual differences we pooled all the hits and misses trials from all individuals.

***Influences of the power of ongoing oscillations.*** For each electrode and each point of the time-frequency plane we computed the power median difference between the 752 hits and the 837 misses and the corresponding *p*-value using the ranksum test. To robustly assess the significance of the results we applied a resampling method to electrodes showing significant differences (*p* < 0.001) in power during the pre-stimulus period according to the ranksum test. In the resampling we assumed as a null hypothesis that that hits and misses are identical. We randomly draw 752 trials from the total 1589 trials over the 12 subjects and assigned them to the hits category and the remaining trials to the misses. The corresponding pseudo-statistics (median difference) were then computed for each bin in the time-frequency plane. We finally computed the probability that a pseudo-statistic was more extreme than the actual one. The number of permutations done was 200 and the significance level for the permutation test set to α = 0.001.

***Influences of the phase of ongoing oscillations.*** For the analysis of the phase difference, the phase bifurcation index (PBI) between hits and misses trials was computed for each point of the time-frequency plane (Busch et al., 2009). The PBI is bounded between −1 and 1 with one representing perfect phase locking in both conditions [see (Busch et al., 2009)] but at opposite phase angles. Positive PBI values are observed when phases of the ongoing oscillations are locked to different phase angles for hits and misses and negative values when only one condition exhibit phase locking. As in Busch et al. (2009) we assessed the statistical significance of the results using the Watson's two-sample *U*2-test under the null hypothesis of random phase distributions for hits and misses (PBI value close to zero). As for the power, we further carried permutation tests to increase the statistical power on the PBI statistic with the significance level set to α = 0.001 after 200 resamplings.

## Results

### Dark noise concerns half of the investigated population

**Figure 3:**
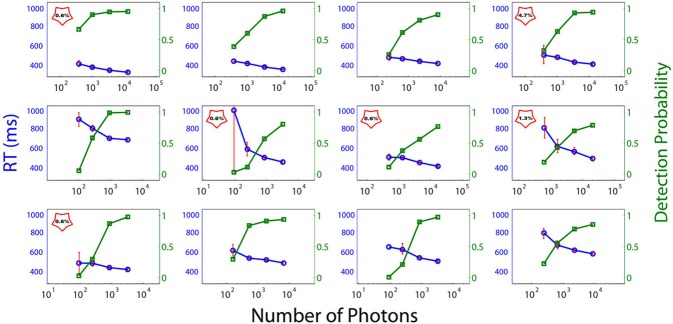
**Psychometric curves (red) and reaction times (blue) of each subject**. Individual RTs and frequency of seeing curves as a function of the number of photons in the light beam (abscissa). The left and right ordinates depict the RTs and the proportion of perceived flashes respectively. No point is depicted in the graph if the number of flashes reported by the subject as perceived is identically zero when no flash was sent. DN subjects are signaled with a red star, which contains the false-positive rate.

Figure 3 depicts the individual RTs and FoS curves as a function of the number of photons sent. The abscissa corresponds to the number of emitted photons and the left and right ordinates to the RTs and the proportion of perceived flashes respectively. Subjects were classified into two categories: DN Subjects (DN), i.e., subjects with Dark-Noise or non-zero false alarm rate and NDN subjects i.e., without Dark Noise. In the plots DN subjects are indicated with a red star containing the false negative rate. Importantly, we cannot exclude that NDN subjects could indeed have shown false positive trials if more flashes would have been presented. Thus, this classification is to be understood more as a gradual ordering than a dichotomous classification.

As can be seen from Figure 3, exactly half of the participants in the experiment reported seeing flashes when no light was emitted, while the other half showed no evidence of dark noise. Consequently, on the basis of the reduced sample of subjects considered here (*N* = 12), we have to conclude that the probability of observing Dark Noise in a population of young healthy controls is exactly 0.5 and therefore significantly different from zero (binomial test, *p* = 0). This criterion allows for a natural splitting of the subjects according to their detection threshold. We cannot, however, discard the possibility that all subjects might exhibit Dark noise if the number of trials increases.

### Frequency of seeing (accuracy) and QE

**Figure 4:**
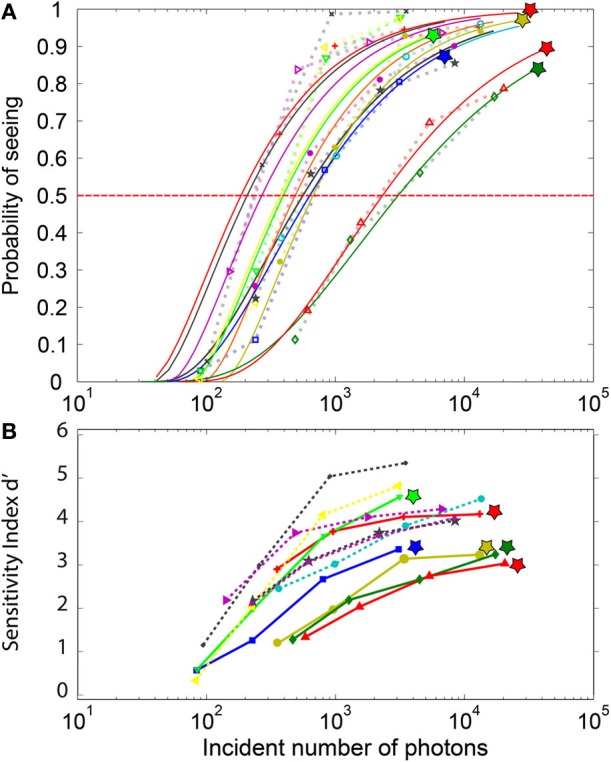
**Psychometric curves for the individual subjects**. **(A)** The FoS curves and their parametric fitting: The symbols represent actual data, while the continuous lines depict their respective log-poisson fits. Each subject is represented by a different color. These fits are used to estimate the minimal number of incident photons required to elicit perception in at least 50% of the trials. The log-poisson distribution allows a reasonable fit for all subjects, even if for a few subjects a linear Poisson distribution leads to a better fit. The stars at the top of the curves indicate subjects with non-zero false-positive rate. **(B)** d-prime sensitivity index for each subject. DN Subjects (indicated by a star) with non-zero false-positive rate (plain lines) tend to have a lower d-prime than the NDN subjects. Yet, two of the NDN subjects are between the best observers.

The estimated number of incident photons necessary to elicit conscious perception determined from the log-Poisson fits to the frequency of seeing curve (Figure 4A) considerably varied across participants (mean: 815, min 181, max: 3051, std: 903) for a threshold set at 50%. Once these values were corrected by the 0.08 factor estimated by Hecht we obtained: mean: 65, min 17, max: 244, STD: 72. The last subject, requiring 244 photons, reported considerable visual fatigue during the experiment.

The statistical comparison of the DN and NDN subjects in terms of the minimal number of photons required to elicit conscious perception at 50% of the trials revealed no significant differences (parametric *t*-test, *p* = 0.14, non-parametric test rank sum, *p* = 0.33). Indeed, assuming that only 8% of the emitted photons reach the retina we estimated that DN subjects require on average 54 photons to elicit perception in 50% of the trials while NDN subjects require around 35 photons.

### Sensitivity index (d′)

Contrarily to the FoS curve which basically reflects the hit rates, the sensitivity index (Figure 4B) also reflects the false alarms. A d′ value close to 0 describes near chance-level discrimination, values close to 1 are indicative of moderate performance and d′ values above 4 correspond to near optimal performances. As expected, the d′ index depicted in Figure 4B shows a dependency with the number of incident photons which is similar to the one in the FoS curve (Figure 4A). Moreover, the sensitivity index for the DN subjects indicated by the stars is smaller than for the NDN subjects. A two-sample *t*-test on the d′ indeed revealed significant differences (*p* < 0.05) between NDN and DN subjects for the highest intensities (I3 and I4) as shown in Table 1. This difference is indicative of the trade-off between specificity and sensitivity of a subject. As indicated by this measure, DN subjects are more prone to report false percepts but they are as accurate as NDN subjects in detecting similar number of photons. Consequently, NDN subjects are apparently adopting a more relaxed decision criterion than DN subjects with little impact on their performance.

**Table 1:** **Comparison of mean sensitivity index between DN and NDN subjects**.

**Intensity**	**DN subjects**	**NDN subjects**	***p*-value**
I1	1.34	1.76	0.4
I2	2.22	3	0.08
I3	3.17	4.08	0.02
I4	3.59	4.49	0.02

Statistical analyses were performed using parametric two-sample t-test.

### DN subjects are significantly faster to take decisions than noiseless subjects

**Figure 5:**
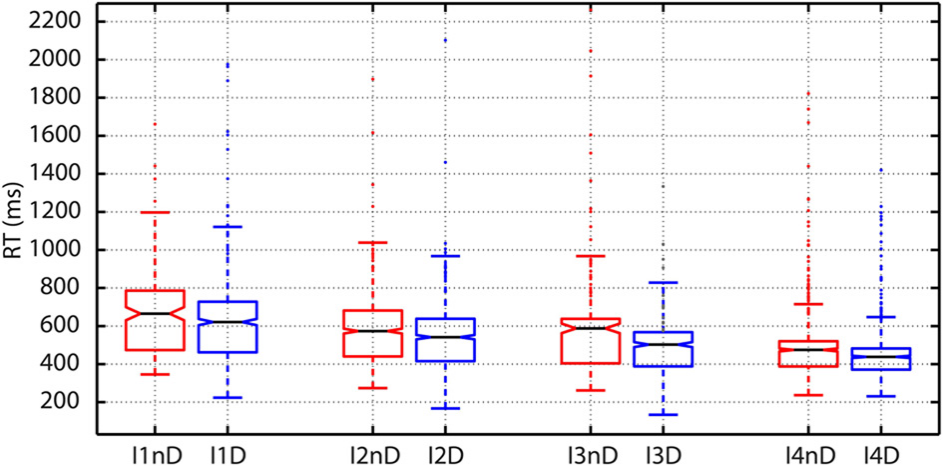
**Variations in reaction time (RT) as a function of the number of photons and the decision threshold: To create the boxplots subjects are divided into two groups according to the presence (DN) or absence (NDN) of false detection (dark noise)**. The RTs of DN subjects (blue boxes) are significantly lower than those of NDN subjects for all intensities (abscissas) since the notches do not overlap. DN subjects seem to trade-off speed and accuracy.

As argued before, in perceptual choice tasks as this one, experimental evidence indicate that choices are made when the firing rate of selective cortical neural population reach a threshold. RTs therefore correlate with the time needed to reach the threshold that is dependent on the difficulty of the choice. This effect was clearly observed in our data. The plot of RTs as a function of the intensity of the flashes showed a clear decrease in RTs for increasing intensities at both, the individual (Figure 5) and population level.

As shown in Table 2, the comparison between the distribution of RTs between subjects with and without dark noise revealed significant differences for all the analyzed intensities. DN subjects were significantly faster to take decisions than NDN subjects. Differences between groups were considerable and varied from 58 ms for the lowest intensity to 100 ms or more for all the others. The rank sum test, as any non-parametric method, is robust to skewed distributions as it makes no assumption about the underlying distribution. In addition, the Lillietest revealed no significant deviations in this dataset from the normality assumption indicating that our RT distributions were not skewed.

**Table 2:** **Comparison of mean reaction times between DN and NDN subjects**.

**Intensity**	**Mean RT**	**Mean RT**	***P*-value (parametric test, *t*-test)**	***P*-value (non-parametric) test, rank sum)**
	**DN**	**NDN**		
I1	587	645	0.03	0
I2	502	621	0	0
I3	474	573	0	0
I4	437	541	0	0

Statistical analyses were performed using parametric (t-test) or non-parametric tests (rank sum). P-values lower than 10^−6^ are marked as 0.

### EEG results (event related potentials, ERPs)

**Figure 6:**
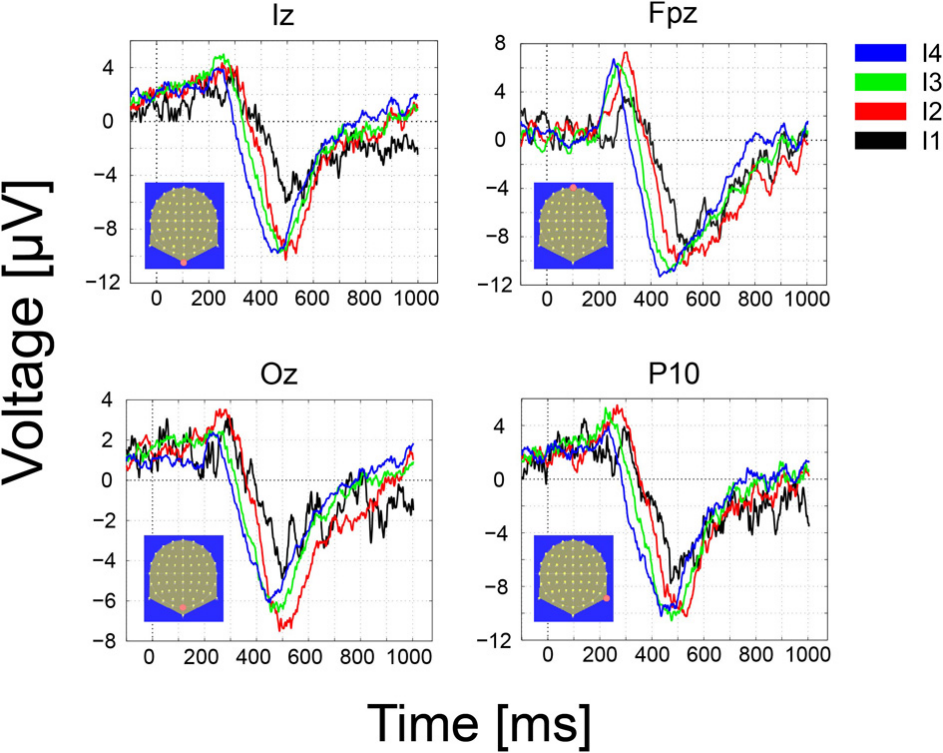
**Grand-Mean ERPs over occipital (Iz, Oz), middle frontal (FPz) and parietal contacts as a function of the number of photons (the four effective light intensities)**. Responses are ordered from the strongest to the weakest intensity (I1–I4) and the following color convention is used: I4, black; I3, red, I2, green, I1, blue. Note that the onset, latency and amplitude of the first ERP component recoded over frontal electrodes varies as a function of the intensity of the stimulus, i.e., the stronger intensity in black peaks earlier than the other intensities. Delays in neural responses across intensities occur earlier over frontal and parietal electrodes. The strongest intensities (I4, black) lead to the fastest response and the weakest (I1, blue) to the slowest.

The GM ERPs for the hit trials and the four different intensities are shown in Figure 6. From the figure, it can be readily seen that the early ERP components (N70 and P100) characteristic of visual ERPs in the presence of visible stimuli are absent over contacts placed over primary visual areas, e.g., electrodes Iz and Oz. Moreover, the first significant (*p* < 0.01) deviations in the GM amplitude with respect to a baseline period of 200 ms (*z*-scores ≥ 2.5 standard deviations away from the baseline mean) were observed first at frontal (Fpz, 211 ms for the strongest intensity), and slightly later for Occipital electrodes (Iz, Oz, 251 ms for the strongest intensity). In the misses trials (not shown) we observed just marginal deviations from the prestimulus baseline that disappeared after corrections for multiple tests.

The onset, latency and amplitude of the first ERP component recorded over frontal electrodes varied gradually as a function of the intensity of the stimulus. The stronger stimuli (black trace) peaked first than the other intensities. The delay across intensities was more clearly observed on the second, negative ERP component peaking between 400 and 600 ms and appeared over frontal, parietal and occipital electrodes.

**Figure 7:**
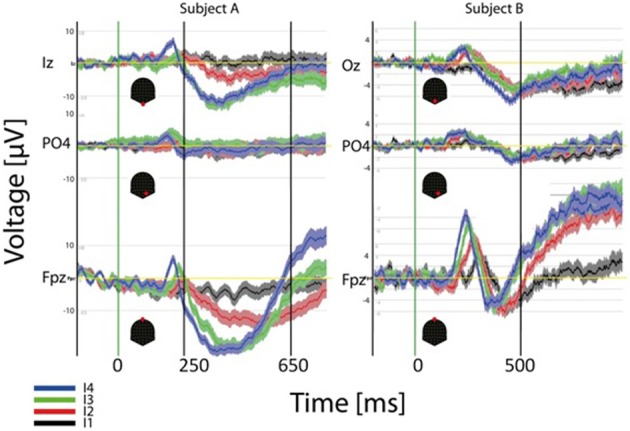
**Individual (2 subjects) ERP averages for the four intensities at occipital, parietal and frontal electrodes**. The highest intensities show faster response at the three sites but effects are more pronounced over frontal contacts.

ERP results at the single subject level are illustrated in Figure 7. In this case the ERP for the four different intensities (dark thick traces) and the standard error around the mean are depicted for three channels and two different subjects. Each subject is shown in a different column. As observed for the GM data peak delays across intensities are obvious over frontal contacts (FPz) and much smaller or inexistent over parietal and occipital contacts. Polarities and latencies of the components are similar to those observed for the GM data. Note that occipital responses for the lowest intensity (black trace) are absent at the initial processing stages suggesting that a critical mass of activation of the primary visual cortex necessary to produce the ERP components is absent. Note also the considerable variability across these subjects for the duration of the second negative component. The RTs for these two subjects for the strongest intensity are 640 ms (left subject) and 503 ms (right).

No significant early (before 300 ms) differences between the ERPs of DN and NDN were observed. The earliest significant differences started around 380 ms after stimulus onset and lasted for around 400 ms over several frontal, central and parietal contacts. Given the significant differences in RTs between DN and NDN subjects and the fact that significant differences coincide with or slightly precede the motor responses we consider risky to interpret these differences in terms of post-retinal decision processes. The significant differences observed might be due to pre-motor and motor events which are likely to occur earlier in DN subjects given their significantly short RTs. Yet, the lack of early significant differences between observers relying on different decision criteria is at odds with the idea that decision criteria are already set during the earliest stages of visual processing.

**Figure 8:**
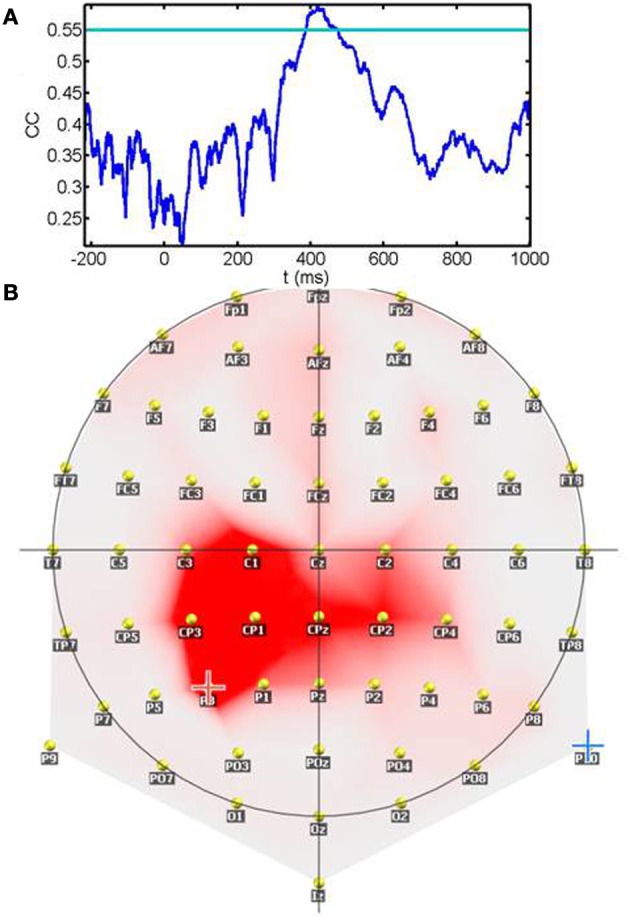
**Correlation between the sensitivity index (d′) and the ERP amplitude across subjects: (A) shows the temporal profile of the correlation coefficient between sensitivity and ERPs amplitudes**. The plot represents the maximum correlation detected irrespective of spatial location (electrode). The horizontal blue line depicts the significance level after multiple tests correction. Note that correlations become significant in the late processing period preceding motor responses. **(B)** Spatial location of the contacts showing significant correlations during the 400–480 ms window. Rather than scattered in space correlations between sensitivity and ERP amplitude are clustered around left parietal and centro-parietal contacts with the maxima placed over P3.

Figure 8A shows the temporal profile of the correlation coefficient between sensitivity and ERPs amplitudes. The plot represents the maximum correlation detected irrespective of spatial location (electrode). Significant correlations (*p* < 0.01) after correction for multiple tests are values surpassing the horizontal blue line. As seen from the plot, correlations are below significance during the 200 ms prestimulus period and the early period that comprises the initial 400 ms. Correlations become significant around a 100 ms temporal window starting at 400 ms. The spatial location of the contacts showing significant correlations during the 400–480 ms window is represented in panel 8B. Rather than scattered in space correlations between sensitivity and ERP amplitude are clustered around left parietal and centro-parietal contacts with the maxima placed over P3. In summary, sensitivity correlates with neural responses during a relatively late post-stimulus period that precedes the timing of motor responses and is observed over contacts relatively distant from primary visual areas.

### EEG results (pre-stimulus differences)

#### Differences in the power of ongoing oscillations between hits-misses

We compared for each electrode the spectral power between hits and misses trials for each frequency bin on a time window of 2.5 s around the stimulus (−1.5 to 1 s). Coinciding with previous reports in the literature (Busch et al., 2009) we observe a significant pre-stimulus enhancement in the power of alpha oscillations (6–8 Hz) over occipital (Oz/Iz) and centro-medial contacts (Fz) for misses (unseen) trials. Differences started earlier (around 500 ms before stimulus) onset over the occipital contacts and slightly later (250 ms before stimulus) over frontal contacts. The significantly increased power for ongoing oscillations in unseen trials lasted until ~20 ms after flash onset.

#### Differences in the phase of pre-stimulus ongoing oscillations between hits-misses trials

To test for consistent pre-stimulus differences in the phase of on-going pre-stimulus oscillations between hits-misses trials we relied on the concept of PBI previously used by (Busch et al., 2009) for similar purposes on a similar experimental context. We found significant negative PBI at occipital and prefrontal sites for a very low frequency band centered around two Hz within a long interval starting around one second before stimulus onset and lasting until 500 ms after the stimulus (not shown). Such highly significant negative PBI values are reasonable in the post-stimulus period since they can reflect that ERPs are evoked in only one of the conditions analyzed (e.g., hits) as was the case in our data. Yet, the pre-stimulus differences in this low frequency band could be a consequence of the extremely low temporal resolution at lowest frequencies imposed by the time-frequency resolution trade-off which is inherent to any time-frequency decomposition.

**Figure 9:**
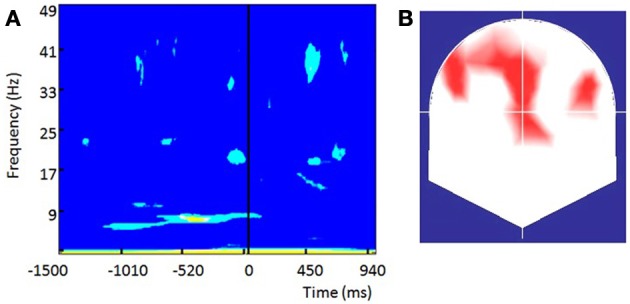
**The phase of prestimulus ongoing oscillations influences the visual detection threshold long before retinal processing is initiated: (A) Phase bifurcation index (PBI) across all subjects for channel Fz**. Only significant values are shown (yellow, *p* < 0.01), light blue (*p* < 0.05). Positive PBI values indicate that phase distributions are locked to different phase angles for hits and misses. PBI shows the strongest significance within the alpha band (~8 Hz) within the period from −500 to −250 ms preceding stimulus onset. **(B)** Topographical representation of contacts showing significant (*p* < 0.05) PBI values at 8.4 Hz within the pre-stimulus interval from −450 to −250 ms preceding stimulus onset.

Highly significant positive PBI values (Figure 9A) were observed within the alpha band (centered at ~8 Hz) over frontal and fronto-central contacts with two small clusters over left and right frontal electrodes (Figure 9B). In similarity to Busch (Busch et al., 2009), differences in the pre-stimulus phase were more pronounced over the Fz contact (shown in Figure 9A). Significant differences started earlier than in Busch study (around 1 s before stimulus onset) and lasted until 20 ms after the stimulus. The most significant differences within the alpha band were observed within the pre-stimulus interval ranging from −500 to −250 ms and therefore slightly earlier than the −300 to −100 window reported by Busch. Yet, significant differences in our study were much more sustained. While some significant differences in positive PBI values were seen within the beta and gamma bands shortly before the stimulus onset their significance was lower than the effects observed within the alpha band. The fronto-central topography of the alpha band differences is remarkably similar to the one observed by Busch.

## Discussion

In this study, we investigated the QE of the human visual system using strictly controlled visual pulses of unusually short duration (~1 ms). By the combined use of psychophysical approaches and neural data we aimed to clarify: (1) the sources affecting the QE and its inherent across trial variability, (2) the divergence between experimental (in animal) and behavioral (in humans) estimates of QE, and (3) the origin (retinal or post-retinal) of processes limiting behavioral sensitivity. Flashes of light, with a controlled probabilistic distribution of photons were sent into the retina of 12 dark-adapted subjects who were prompted to indicate if they perceived or not a light. EEG and RTs were measured along the experiment using a very high frequency sampling (1024/2028 Hz) and a relatively high spatial sampling (64 channels).

The detection threshold, i.e., the number of photons required to trigger a conscious percept, was determined from the FoS curve (Hecht et al., 1942) by measuring the fraction of trials in which a flash was reported as perceived as a function of the number of photons incident at the cornea. Despite considerable interindividual variability, we concluded that on mean around 70 photons are required for untrained subjects to trigger perception 50% of the time. This estimate is considerably high in view of the reliability demonstrated by rods to detect single photons (Rieke and Baylor, 1998). Consequently, and in agreement with previous experiments, the QE estimated from human behavior is very low compared with the absorptive QE estimated from the properties of light photoreceptors at the retina. Either much more photons are required to elicit conscious perception than to elicit responses in photoreceptors or, as commonly argued in the vision literature, the existence of dark noise increases the rate of false-positive forcing observers to adjust the detection threshold and decreasing sensitivity.

The use of psychophysical and neurophysiological measures added to the FoS curve allowed to shed further light on the origins of the dark noise. Indeed, exactly half of the subjects in our sample reported perception in trials where no flashes were presented (Dark Noise). As argued in (Rieke and Baylor, 1998) the dark noise might be the consequence of Poisson fluctuations in photon absorption at the level of the retina. Indeed, continuous noise in mammalian rods can generate fluctuations that look very much like true photon responses (Baylor et al., 1984). Following the reasoning of Barlow (Barlow, 1956; Hallett, 1969) differences in the false-positive rate across observers should arise from inter-individual differences in detection threshold. Consequently, more sensitive subjects, i.e., subjects detecting more photons, should be more prone to detect flashes when there is no one. Yet, while we observed differences in sensitivity between DN and NDN observers we identified no significant differences in the minimal amount of photons detected by each group. DN observers were worst than NDN observers because of their lower sensitivity to specificity ratio *as reflected by the d' measure*. However, two of the DN subjects ranked among the best observers in terms of sensitivity (see Figure 3). This result suggests than other factors beyond purely retinal processing might intervene in lowering visual sensitivity and detection thresholds.

In agreement with this idea we observed clear differences in the response speed of both groups of observers. The DN group of observers, prone to report non-existing flashes, was significantly faster than the NDN group to report true flashes. This indicates that the DN group compromised accuracy to gain in response speed. Despite the fact that no overt instruction about response speed was given to participants, the long and boring nature of the experiment might have played a motivational role in maximizing speed. Therefore, the decrease in detection threshold for the DN observers might, at least in part, be due to motivational factors instead of statistical fluctuations in photon absorption in retinal photoreceptors. This view is supported by the fact that DN subjects have a significantly lower senstitity index d′ indicative of a more relaxed decision criterion.

Additional evidences for non-retinal contributions to sensitivity/detection thresholds were obtained from the analysis of EEG data. First, the comparison of the ERP data between DN and NDN observers failed to detect any significant differences in the early period where the basic visual processing on primary visual areas is known to occur. This can indicate that the decision criteria is set later in the chain of neural processing and it is therefore not established on purely retinal basis. However, this result could be also explained in terms of true differences at the level of primary visual areas that are too weak to be detected by scalp electrodes. Second, the essay to correlate the sensitivity index across observers with the amplitude of the ERP components failed again to reveal early correlations. Importantly, the correlation analysis is less affected by the strength of the visual responses. In fact, significant correlations between the sensitivity index of the different individuals and the amplitudes of the ERP signals were observed in the interval preceding motor responses over parietal contacts. Both the location of the contacts and the timing of the differences, suggest that interindividual differences in sensitivity are linked to post-retinal decisional processes. Finally, the analysis of prestimulus states oriented to understand the sources of the large inter-trial variability in detection threshold within the same observer clearly indicate a role for extra-retinal processes likely linked to motivational factors. We indeed observed significant differences between perceived and unperceived dim flashes in terms of the amplitude and phase of neural oscillations long before the onset of the flash. Since no retinal processing is ongoing during the pre-stimulus period we need to accept the conclusion that the visual threshold is not exclusively set by Poisson fluctuations at retinal photoreceptors. While the rods might behave as exquisite and reliable photoreceptors able to react to single photons, there are additional mechanisms preventing the access of the information transmitted by the rods to conscious perception. Access to conscious perception is required for a decision to occur which ultimately leads to the overt motor response necessary to build the FoS curve.

How can motivational factors, reflected by pre-stimulus ongoing oscillations, determine the fate of photons that are reliably processed by rod photoreceptors? Could such pre-stimulus mechanism ultimately decrease the efficiency of the whole visual detector composed by the retina and the brain? In practice, living systems need to integrate and adapt processing of the responses of their sensory systems according to rapid fluctuations in basic motivational demands, e.g., hunger or predators. To do so, the whole system must ignore the incessant flow of “reliable” sensory information from every single sensory modality to favor urgent motivational needs as to prevent the bottleneck in central processing resources and limited motor resources. Accumulating evidence suggests that attention selectively synchronizes the rhythmic responses of those neurons that are tuned to the spatial and featural attributes of the attended sensory input. The strength of synchronization is functionally related to perceptual accuracy and behavioral efficiency (Womelsdorf and Fries, 2007). Motivation seems to drive attentional circuits as to amplify responses within the interesting sensory modality so that they become competitive for the limited amount of motor resources available to living systems. On this line of reasoning, it is very reasonable to observe the largest correlates of psychophysical parameters such as sensory and decision thresholds at the latest processing stages and very close in time to the motor responses. While the overall efficiency of individual sensory modalities seems to decrease with such pre-stimulus filtering mechanisms they are required to cope with multisensory processing and limited motor resources. Undoubtedly, further studies are required to strictly quantify the relative influence of statistical fluctuations in retinal photon absorption and that of motivation on setting the absolute threshold in human vision. This study, which reproduces one of the classical experiments addressing the fundamental physical limits of living sensory systems, will hopefully help to promote a more integrative view on how the boundaries of multisensory systems competing for limited resources are set above the limits of each individual modality.

### Conflict of interest statement

The authors declare that the research was conducted in the absence of any commercial or financial relationships that could be construed as a potential conflict of interest.
